# Supplementary material for: “If I tell you my problems, how will you perceive me?”: A qualitative study of mental health knowledge, barriers, and opportunities for care among Kenyan adolescents during COVID-19
Source: PLoS One. 2025 May 23;20(5):e0323907. doi: 10.1371/journal.pone.0323907 (PMC12101854; doi:10.1371/journal.pone.0323907)
Supplement: Attachment — Submitted filename: Rebutal letter PONE.docx [file pone.0323907.s002.docx]

31^st^ March 2025

To

The Editor

PLOS ONE

Dear Editor,

**Re**: *Resubmission of paper* PONE-D-24-44355 “If I tell you my problems, how will you perceive me?”: A Qualitative Appraisal of Mental Health Knowledge, Barriers, and Opportunities for Care among Kenyan Adolescents

We want to thank you for reviewing our manuscript. We are grateful to the reviewers for their comments and feedback. Below is a point-by-point response.

We hope the edited paper will meet your expectations.

Regards

1. Please ensure that your manuscript meets PLOS ONE's style requirements, including those for file naming. The PLOS ONE style templates can be found at

https://journals.plos.org/plosone/s/file?id=wjVg/PLOSOne_formatting_sample_main_body.pdf and

https://journals.plos.org/plosone/s/file?id=ba62/PLOSOne_formatting_sample_title_authors_affiliations.pdf

**Response**: Thank you, we have done this

2. We note that the grant information you provided in the ‘Funding Information’ and ‘Financial Disclosure’ sections do not match.

When you resubmit, please ensure that you provide the correct grant numbers for the awards you received for your study in the ‘Funding Information’ section.

**Response**:

3. Thank you for stating the following financial disclosure:

“Research reported in this publication was supported by the Fogarty International Center of the National Institutes of Health under Award Number K43TW010716, which also supported the contributions of MK to this work. The content is solely the responsibility of the authors and does not necessarily represent the official views of the National Institutes of Health. The qualitative inquiry is part of an embedded study protocol for the Measurement of Mental Health of Adolescents at population level (MMAP) led by UNICEF and the inquiry reported here was partially supported by UNICEF Innocenti office.”

Please state what role the funders took in the study. If the funders had no role, please state: "The funders had no role in study design, data collection and analysis, decision to publish, or preparation of the manuscript." If this statement is not correct you must amend it as needed.

Please include this amended Role of Funder statement in your cover letter; we will change the online submission form on your behalf.

**Response**:

4. Please note that funding information should not appear in the Acknowledgments section or other areas of your manuscript. We will only publish funding information present in the Funding Statement section of the online submission form. Please remove any funding-related text from the manuscript.

**Response**:

5. We note that you have indicated that there are restrictions to data sharing for this study. For studies involving human research participant data or other sensitive data, we encourage authors to share de-identified or anonymized data. However, when data cannot be publicly shared for ethical reasons, we allow authors to make their data sets available upon request. For information on unacceptable data access restrictions, please see http://journals.plos.org/plosone/s/data-availability#loc-unacceptable-data-access-restrictions.

Before we proceed with your manuscript, please address the following prompts:

a) If there are ethical or legal restrictions on sharing a de-identified data set, please explain them in detail (e.g., data contain potentially identifying or sensitive patient information, data are owned by a third-party organization, etc.) and who has imposed them (e.g., a Research Ethics Committee or Institutional Review Board, etc.). Please also provide contact information for a data access committee, ethics committee, or other institutional body to which data requests may be sent.

**Response**: There are no ethical or legal restrictions on sharing data set. Our study received ethical approval from Kenyatta National Hospital-University of Nairobi Ethics review committee (https://erc.uonbi.ac.ke/ ), whose contact is uonknh_erc@uonbi.ac.ke

b) If there are no restrictions, please upload the minimal anonymized data set necessary to replicate your study findings to a stable, public repository and provide us with the relevant URLs, DOIs, or accession numbers. Please see http://www.bmj.com/content/340/bmj.c181.long for guidelines on how to de-identify and prepare clinical data for publication. For a list of recommended repositories, please see https://journals.plos.org/plosone/s/recommended-repositories. You also have the option of uploading the data as Supporting Information files, but we would recommend depositing data directly to a data repository if possible.

**Response**: Thank you, we have uploaded our anonymized data as Supporting information file.

Please update your Data Availability statement in the submission form accordingly.

**Response**:

6. In this instance it seems there may be acceptable restrictions in place that prevent the public sharing of your minimal data. However, in line with our goal of ensuring long-term data availability to all interested researchers, PLOS’ Data Policy states that authors cannot be the sole named individuals responsible for ensuring data access (http://journals.plos.org/plosone/s/data-availability#loc-acceptable-data-sharing-methods).

Data requests to a non-author institutional point of contact, such as a data access or ethics committee, helps guarantee long term stability and availability of data. Providing interested researchers with a durable point of contact ensures data will be accessible even if an author changes email addresses, institutions, or becomes unavailable to answer requests.

Before we proceed with your manuscript, please also provide non-author contact information (phone/email/hyperlink) for a data access committee, ethics committee, or other institutional body to which data requests may be sent. If no institutional body is available to respond to requests for your minimal data, please consider if there any institutional representatives who did not collaborate in the study, and are not listed as authors on the manuscript, who would be able to hold the data and respond to external requests for data access? If so, please provide their contact information (i.e., email address). Please also provide details on how you will ensure persistent or long-term data storage and availability.

**Response**: Our study received ethical approval from Kenyatta National Hospital-University of Nairobi Ethics review committee (https://erc.uonbi.ac.ke/ ), who can be contacted on email via uonknh_erc@uonbi.ac.ke

7. When completing the data availability statement of the submission form, you indicated that you will make your data available on acceptance. We strongly recommend all authors decide on a data sharing plan before acceptance, as the process can be lengthy and hold up publication timelines. Please note that, though access restrictions are acceptable now, your entire data will need to be made freely accessible if your manuscript is accepted for publication. This policy applies to all data except where public deposition would breach compliance with the protocol approved by your research ethics board. If you are unable to adhere to our open data policy, please kindly revise your statement to explain your reasoning and we will seek the editor's input on an exemption. Please be assured that, once you have provided your new statement, the assessment of your exemption will not hold up the peer review process.

**Response**: this is a qualitative data which can potentially be used to identify participants if several alternations are not made. We prefer not to share interview transcripts open-access but the senior author can provide upon reasonable request when contacted via email.

8. Your ethics statement should only appear in the Methods section of your manuscript. If your ethics statement is written in any section besides the Methods, please delete it from any other section.

**Response**: Thank you, we have deleted it from elsewhere and retained it in the methods section only

9. Please include a separate caption for each figure in your manuscript.

**Response**: Thank you, we have included figure captions (please see pages 5 and 8)

10. Please review your reference list to ensure that it is complete and correct. If you have cited papers that have been retracted, please include the rationale for doing so in the manuscript text, or remove these references and replace them with relevant current references. Any changes to the reference list should be mentioned in the rebuttal letter that accompanies your revised manuscript. If you need to cite a retracted article, indicate the article’s retracted status in the References list and also include a citation and full reference for the retraction notice.

**Response**: Thank you, we have reviewed the references

Review Comments to the Author

**Reviewer #1**: It is an interesting topic; "If I tell you my problems, how will you perceive me?”: A Qualitative Appraisal of Mental Health Knowledge, Barriers, and Opportunities for Care among Kenyan adolescent's ."

1. Abstract:- please remove the discssion part from the abstract section. No need of mentioning it in the abstract.

**Response**: Thank you for this suggestion, we have removed the discussion from the abstract

2. Methodology:- You have used a qualitative methods, but please specify which type of qualitative study design/method you have used in your study?

**Response**: this is an anthropologically embedded cultural validation of mental health problems and measurement tools study. Such studies aim to address culturally responsive approaches to addressing measurement of mental health problems.

3. Ethical clearance Vs Ethical approval:- Please not to write the ethical clearance in two sections, i.e remove the ethical clearance secthon. Additionaly, there are two different ethical approval number; Kenyan national commission for Science, Technology and innovation NACOSTI/P/19 and NACOSTI/P/8757. Which one is your correct approval No.??

**Response**: We have removed the section of ethical clearance and replaced it with ethical approval. We apologize for the oversight, the correct NACOSTI number is NACOSTI/P/19/77705/28063

**Reviewer #2: Reviewer comment and suggestions**

Generally congratulation to the authors for the interesting paper which informed the health of adolescent and their caregiver however there is several issues need to improve.

Adhere to journal guideline in organizing the work

Title

• Your title is understandable but could be clearer and more structured also qualitative appraisal is fine but specifying ‘’A QUALITATIVE STUDY ‘’makes it more formal.

**Response**: Thank you for pointing to this, we have now revised it to “A Qualitative Study” per your suggestion

Abstract

• On this part the background/introduction it’s very narrow the authors should revise and improve and I noted the (LMICs) the ways explain its not formal revise.

**Response**:

• There is no need to write the discussion on part of abstract

**Response**: Thank you, we have removed the discussion part from the abstract

• I would ask the authors how many population in this study?

**Response**: Thank you for pointing this out; however, in the materials and methods section of the abstract, we recorded that our study population was 46 participants, consisting of adolescents and their caregivers.

INTRODUCTION

• I did not understand COVID-19 come from the author should be more specific the reader will confuse revise and start from the title.

**Response**: Thank you, we have now included COVID-19 in the title per your suggestion to make it clear

METHOD /MATERIAL

• I noted on part of method and material the authors write (QUALITATIVE METHODOLOGY) revise and improve

**Response**: we have made tweaks to the sections making it more specific.

• How trustworthiness was assured?

**Response**: For confidentiality and trustworthiness, the participants were given identifying numbers, and did not say their names throughout the FGDs. Recorded FGDs and transcripts were saved in password-protected computers, accessible to research team members only. In this way, no participant's identifying information was accessed by anyone outside the research team.

DISCUSSION

• Well written but the authors should remove this word (Overall summary findings)

**Response**: Thank you for this suggestion, we have now removed the sub-heading

Reference

• Several references do not fit the requirements of Vancouver style. Revise and improve them.

**Response**: We have now revised the references and improved them
